# Fludarabine/Cyclophosphamide Conditioning Regimen in Aplastic Anemia Patients Receiving Matched-Sibling Donor Transplant Is Non-inferior to ATG/Cyclophosphamide: A Single-Center Experience from Pakistan

**DOI:** 10.1155/2022/1442613

**Published:** 2022-09-09

**Authors:** Uzma Zaidi, Mushkbar Fatima, Shafaq Abdul Samad, Kashif Shafique, Hira Fatima Waseem, Tasneem Farzana, Tahir Sultan Shamsi

**Affiliations:** ^1^Department of Clinical Hematology, National Institute of Blood Diseases & Bone Marrow Transplantation, Karachi, Pakistan; ^2^Department of Research and Development, National Institute of Blood Diseases & Bone Marrow Transplantation, Karachi, Pakistan; ^3^School of Public Health, Dow University of Health Sciences, Karachi, Pakistan

## Abstract

The successful outcome of allogeneic hematopoietic stem cell transplant (HSCT) in aplastic anemia patients is driven by suitable donor selection, appropriate conditioning regimen, early intervention, and optimal supportive care after transplant. Pakistan, being a developing country, faces grave economic challenges due to meager health care budget; therefore, cost constraints remain the foremost impediment in optimizing transplant facilities for socioeconomically deprived patients. We conducted a single-center retrospective analysis of aplastic anemia patients (*N* = 130), who received matched sibling donor transplants from 2011 to 2019, treated with either fludarabine/cyclophosphamide (Flu/Cy) or antithymocyte globulin/cyclophosphamide (ATG/CY) conditioning regimen. Median age was 16 years (IQR, 11-20), and it ranged from 3 to 48 years. The median time from diagnosis to transplant was 3 months (IQR, 2 to 4), and it ranged from 1 to 8 months. The estimated overall survival (OS), relapse-free survival (RFS), and GvHD-free survival (GFS) were found to be 69.0%, 66.7%, and 64.3% in the ATG/Cy group while 76.1%, 72.7%, and 62.5% in the Flu/Cy group, respectively, after a median follow-up of 30 months (IQR, 8 to 55), and it ranged from 0 to 98 months for the study groups. The Flu/Cy regimen was well tolerated and was not associated with increased risk of GvHD. Hence, it may be an appropriate alternative conditioning regimen for developing countries with limited health care resources.

## 1. Introduction

Severe aplastic anemia is a lethal condition with an incidence of 2 to 7cases per million individuals across the globe annually [1]. The prevalence in the Asian population is 2 to 3 times higher as compared to western countries owing to the increased predisposition to environmental contaminants like benzene, arsenic, and pesticides, particularly in the inhabitants in the countryside [2].

In Pakistan, according to an estimate, 3.5 patients/million population are diagnosed with aplastic anemia every year, and less than one-third of them attain cure by bone marrow transplant due to inadequate access to advanced health care [3]. The National Institute of Blood Diseases & Bone Marrow Transplantation (NIBD & BMT), being a tertiary care referral center in the second largest province of Pakistan, caters the maximum number of aplastic anemia cases across the country. The main source of subsidy in our center is hospital welfare, nongovernment organizations (NGOs), and philanthropists. The provincial government has taken an initiative over the last few years to support a finite number of transplants. The only way to defy the economic burden is to customize the local transplant policies by limiting the length of hospital stay and laboratory investigations and altering the conditioning regimens to reduce the drug toxicities to curtail the overall cost of transplant.

Though the combination of antithymocyte globulin and cyclophosphamide (ATG/Cy) conditioning in aplastic anemia continues as the benchmark in matched-sibling donor transplant [4], fludarabine-based conditioning has emerged as a promising alternative regimen with excellent survival and reduced toxicity particularly in adults [5]. Addition of ATG in the conditioning of aplastic anemia substantially increases the overall cost of transplant, owing to the drug cost itself and increased risk of posttransplant infections associated with delayed immune reconstitution and hence prolonging hospitalization and increasing the cost of laboratory monitoring [6].

Data on using fludarabine/cyclophosphamide (Flu/Cy) conditioning in pediatric and adult patients is scarce with the concern of increased rate of graft failure. We used fludarabine in combination with standard dose of cyclophosphamide (200 mg/kg) without adding ATG in severe aplastic anemia patients to minimize the risk of graft failure as well as GvHD.

Here, we present a retrospective analysis of Flu/Cy versus ATG/Cy conditioning in severe aplastic anemia patients who underwent matched-sibling donor transplant at our center. The purpose was to evaluate an influence of an alternative regimen (Flu/Cy) on OS, GFS, and RFS. This was of particular interest because this alternative strategy might improve the overall outcome of patients in developing countries like Pakistan where the effective implementation of stem cell transplant program is a real challenge.

## 2. Material and Methods

### 2.1. Diagnosis and Patient Selection

A single-center retrospective analysis of 130 patients with AA who underwent matched-sibling donor transplant was carried out at NIBD & BMT from January 2011 to December 2019. The study was approved by the Institutional Review Board of the National Institute of Blood Diseases & Bone Marrow Transplantation (NIBD/IRB-218/11-2021). All patients with severe and very severe aplastic anemia who had negative chromosomal breakage analysis for Fanconi's anemia, no evidence of PNH clone, no evidence of MDS-related cytogenetic abnormality, ECOG performance status of 0-1, no major organ dysfunction, and negative viral serology were eligible for transplant. None of the patients had received ATG prior to transplant. Pretransplant assessment included autoimmune and biochemical profile, echocardiography, CT-chest, pulmonary function test, and pregnancy test in females of child-bearing age.

### 2.2. Conditioning Regimen, GvHD, and Antimicrobial Prophylaxis

Patients were divided into 2 subgroups for the purpose of analysis, based on the conditioning regimen. One group received ATG/Cy (Thymoglobulin, 2.5 mg/kg/day × 4 days and cyclophosphamide, 50 mg/kg/day × 4 days), whereas the other group received fludarabine (30 mg/m^2/^day × 4 days and cyclophosphamide, 50 mg/kg/day × 4 days). The ATG-based conditioning was given to patients who had financial subsistence from the provincial government. GvHD prophylaxis consisted of continuous parenteral cyclosporine infusion (3 mg/kg/day) from Day-2 till Day+14 posttransplant, followed by oral cyclosporine (6 mg/kg/day) given in two divided doses daily along with intravenous methotrexate (15 mg/m^2^) on Day+1 and 10 mg/m^2^ on Day+3, +6, and+11, posttransplant. Cyclosporine doses were adapted according to institutional policy to avoid drug toxicities like renal insufficiency, microangiopathy, and neurological complications. Graft source was PBSC and bone marrow harvest. For donors above 12 years of age, only PBSC were used as a graft source, whereas for donors younger than 12 years, bone marrow alone or bone marrow plus PBSC were used as graft sources.

All patients received ciprofloxacin, fluconazole, and acyclovir as part of antibacterial, antifungal, and antiviral prophylaxis, respectively, starting from Day-2 of conditioning. Prophylaxis against Pneumocystis jirovecii was initiated at Day+28 posttransplant after complete recovery of blood counts. For CMV-reactivation, ganciclovir was used as preemptive therapy if >1000 copies/mL were detected and was continued until 2 negative PCR readings were obtained.

Monitoring of CMV and BKV DNA by PCR was carried out once weekly from Day+7 till Day+28 and fortnightly thereafter till Day+100 posttransplant. Therapeutic monitoring for cyclosporine was carried out with a target concentration of 200-300 ng/mL during the first three months posttransplant. Donor chimerism was performed by STR method on Days+28, +60, 120, and +180 and at one year. Irradiated blood components were used in all patients from the start of conditioning therapy. The choice of red cells, plasma, and platelet components was based on ABO compatibility between recipient and donor as described by the American Association of Blood Banking [7].

### 2.3. Definitions of Engraftment and Graft Failure

Neutrophil engraftment was defined as the first of 3 consecutive days with absolute neutrophil count > 0.5 × 10^9^/L. Platelet engraftment was defined as platelet count > 20 × 10^9^/L without platelet transfusion for 7 days. Primary graft failure (PGF) was defined as failure to achieve neutrophil count of >0.5 × 10^9^/L by Day+28. Secondary graft failure (SGF) was defined as subsequent decline in absolute neutrophil count < 0.5 × 10^9^/L after Day+28, not related to relapse, infection, or drug toxicity. Relapse of primary disease was defined as peripheral blood cytopenias and hypocellular bone marrow along with complete loss of donor chimerism, after completion of treatment once the immunosuppression was tapered off. Neutrophil and platelet engraftments were defined according to recommendations of the European Blood and Marrow Transplant Society.

### 2.4. Statistical Analysis

**Figure 1:**
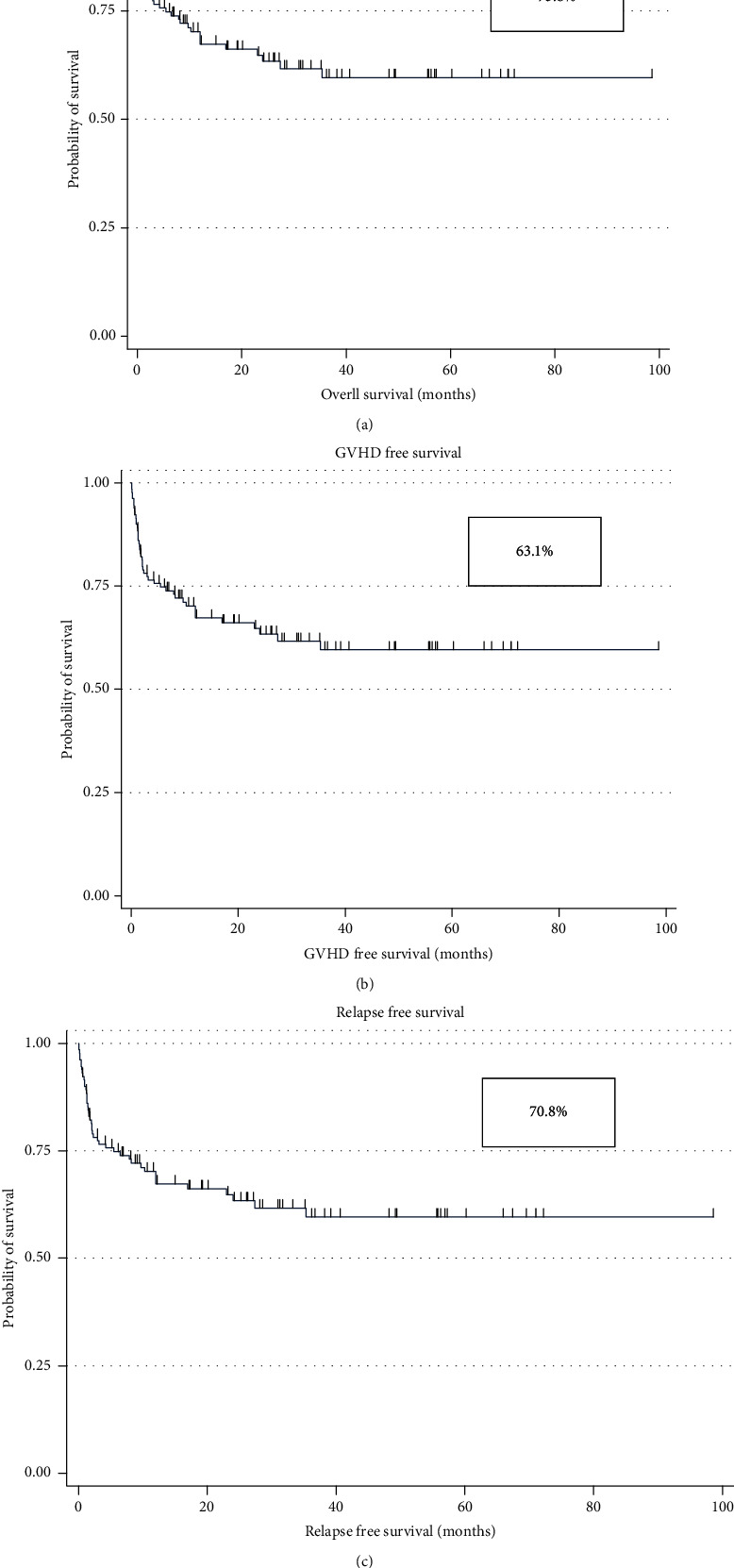
Overall survival (OS), GvHD-free survival (GFS), and relapse-free survival (RFS) in acquired aplastic anemia patients: (a) OS was 73.8%, (b) GFS was 63.1%, and (c) RFS was 70.8%.

**Figure 2:**
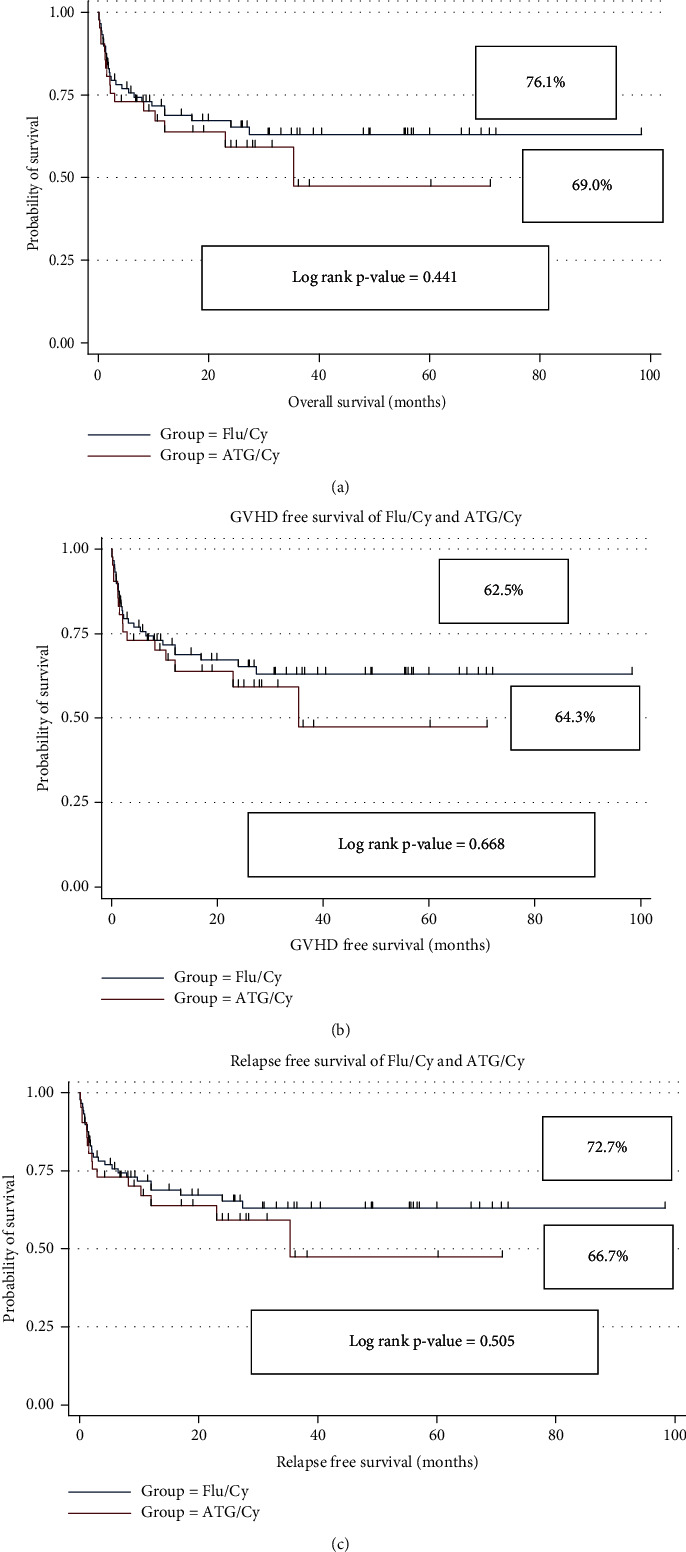
OS, GFS, and RFS as per conditioning regimen in Flu/Cy and ATG/Cy groups.

Data were analyzed by using SPSS version 24. Descriptive statistics for continuous variables were reported as median and interquartile range (nonparametric), while frequencies and percentages were reported for all categorical characteristics (Table 1). The OS was determined by incorporating all the patients who were alive at the time of their last follow-up. The GFS and RFS were calculated by including all the patients who were alive without any evidence of GvHD and relapse at the time of last evaluation. All continuous variables were compared using Mann–Whitney *U* test, whereas Pearson chi-square test and Fisher's exact test were used to assess the association between the two conditioning groups with patient-related categorical variables (Table 2). The Kaplan-Meier method was used to determine the probability of OS, GFS, and RFS (Figure 1). Further, the log rank test was used to assess differences in OS, GFS, and RFS between Flu/Cy and ATG/Cy groups (Figure 2). Univariate analysis was performed to estimate the factors affecting OS, GFS, and RFS (Table 3). Moreover, multivariate Cox regression analysis was applied to find out the significance of different covariates and their effect on different survivals (OS, GFS, and RFS), and hazard ratio with 95% CI was reported (Table 4). A value of *p* < 0.05 was considered statistically significant.

## 3. Results

### 3.1. Patient and Transplant Characteristics

A total of 130 patients fulfilling the inclusion criteria were analyzed in this study. Table 1 depicts the patient and transplant characteristics of the study population. The median age of patients was 16 years (IQR, 11 to 20), and it ranged from 3 to 48 years. The median time from diagnosis to transplant was 3 months (IQR, 2 to 4), and it ranged from 1 to 8 months. The median time to follow-up was 30 months (IQR, 8 to 55), and it ranged from 0 to 98 months for the study groups. Among patients, 68.5% (*n* = 89) were males and 40.8% (*n* = 53) were ≥18 years of age. Female donors to male recipient transplants were 14.6% (*n* = 19). Majority of the patients were categorized as severe aplastic anemia 93.8% (*n* = 122) while 6.2% (*n* = 8) patients had very severe aplastic anemia (VSAA). There were 32.3% (*n* = 42) patients in the ATG/Cy group while 67.7% (*n* = 88) patients in the Flu/Cy group. Eighty-seven percent (*n* = 114) patients were taking Cyclosporine at the time of transplant. The median number of packed red cell transfusions prior to transplant were 3 (IQR, 2 to 5) and single-donor platelet transfusions were 11 (IQR, 3-30). All recipients and donors in this study were CMV seropositive. Donors were 8/8 HLA-matched siblings of either gender. Altogether, 38.5% (*n* = 50) patients underwent ABO mismatch transplants (major, minor, and bidirectional). Stem cell source was GCSF-mobilized peripheral blood (PBSC) in 62.3% (*n* = 81), bone marrow in 26.2% (*n* = 34), and both PBSC and bone marrow in 11.5% (*n* = 15) of cases.

Table 2 describes the transplant characteristics according to the two conditioning groups. The median age of patients was 12 years (IQR, 8-18) in the ATG/Cy group while 17 years (IQR, 12-24) in the Flu/Cy group which indicates that age was slightly higher with a statistically significant difference in Flu/Cy versus ATG/Cy group (*p* < 0.001). The median time from diagnosis to transplant was 3 months (IQR, 2-4) in ATG/Cy, conversely 3 months (IQR, 2-4) in the Flu/Cy group with no statistical difference among the two conditioning groups (*p* = 0.267). The median time from transplant to follow-up was 24 months (IQR, 3-64) in the ATG/Cy group whereas 30 months (IQR, 8-49) in the Flu/Cy group (*p* = 0.813).

### 3.2. Engraftment and Outcome Analysis

The median time to neutrophil engraftment was 12 days (IQR, 10-15), and it ranged from 7 to 35 days, whereas median time to platelet engraftment was 15 days (IQR, 13-20), and it ranged from 9 to 35 days posttransplant. The cumulative incidence of GvHD was 13.8% (*n* = 18), whereas overall incidence of GvHD was 18.2% (*n* = 16) in Flu/Cy versus 4.8% (*n* = 2) in the ATG/Cy group showing significant statistical difference (*p* = 0.038). Acute GvHD was observed in 8.0% (*n* = 7) and 2.4% (*n* = 1) patients in the Flu/Cy and ATG/Cy groups, respectively (*p* = 0.436). Grade I-II acute GvHD occurred in 3.8% (*n* = 5) and grades III-IV in 2.30% (*n* = 3) patients altogether. The incidence of chronic GvHD was 9.1% (*n* = 8) in Flu/Cy versus 4.8% (*n* = 2) in ATG/Cy showing no statistically significant effect on the two groups (*p* = 0.499). 4.6% (*n* = 6) patients had limited stage cGvHD, whereas extensive cGvHD occurred in 3.07% (*n* = 4) patients.

The Kaplan-Meier curve yielded an OS of 73.8%, RFS of 70.8%, and GFS of 63.1% as illustrated in Figure 1. The OS, RFS, and GFS were similar between Flu/Cy (76.1%, 72.7%, 62.5%) and ATG/Cy (69.0%, 66.7%, 64.3%) groups, respectively, with no statistical difference (*p* values = 0.353, 0.403, 0.527, respectively). Primary graft failure (PGF) occurred in 10.0% (*n* = 13), and secondary graft failure (SGF) occurred in 12.3% (*n* = 16) patients. The incidence of PGF was 5.7% (*n* = 5) and 19.0% (*n* = 8) in Flu/Cy versus ATG/Cy groups, respectively (*p* value = 0.027). Five patients with PGF received stem cell boost within first 100 days of transplant; 2 of them were able to restore hematopoiesis and achieved full donor chimerism. Remaining patients with PGF died of infectious causes secondary to severe pancytopenia. Secondary graft failure was observed in 10.2% (*n* = 9) patients in the Flu/Cy group whereas 16.7% (*n* = 7) patients in the ATG/Cy groups (*p* value = 0.392). Six patients with SGF received donor lymphocyte infusions (1-2 doses), out of which 4 patients successfully upheld normal hematopoiesis, whereas 4 patients were treated with eltrombopag and sustained a neutrophil count of >1.0 × 10^9^/L, thus far.

Total 6.9% (*n* = 9) events of disease relapse were observed during the follow-up of patients, out of which 9.1% (*n* = 8) relapses occurred in the Flu/Cy group, whereas 2.4% (*n* = 1) relapses occurred in the ATG/Cy group (*p* value = 0.270). Three patients in the ATG/Cy group underwent second transplant with same donors but switched to Flu/Cy conditioning and maintained a donor chimerism of >90% at 1-year follow-up together with a norm cellular bone marrow.

Table 3 summarizes the univariate analysis of potential risk factors on OS, RFS, and GFS. On applying the chi-square test and Fisher's exact test, no significant association of time to transplant, age, gender, gender mismatch, conditioning regimens, disease category, cyclosporine use, and graft source was found with OS, GFS, and RFS. However, the infused total nucleated cell (TNC) and CD34 count, PGF, and SGF were significantly associated with lower OS, GFS, and RFS in the univariate analyses.

Table 4 shows the multivariate Cox regression analysis performed to estimate the effect of factors like conditioning, CD34 count, TNC count, PGF, and SGF on OS, GFS, and RFS. After adjusting for baseline variables, the use of ATG/Cy versus Flu/Cy conditioning (HR = 0.48, 95% CI: 0.22–1.10; *p* = 0.084) and lower TNC count (HR = 1.97, 95% CI: 0.95–4.07; *p* = 0.066) did not produce any substantial impact on OS in the multivariate analysis. On the contrary, lower CD34 count (HR = 3.18, 95% CI: 1.46–6.94; *p* = 0.003), PGF (HR = 13.22, 95% CI: 5.30–33.02; *p* < 0.001), and SGF (HR = 6.45, 95% CI: 2.74–15.17; *p* < 0.001) evidently increased the risk of mortality. No significant association of ATG/Cy versus Flu/Cy conditioning (HR = 0.47, 95% CI: 0.21–1.06; *p* = 0.072) and lower TNC count (HR = 1.99, 95% CI: 0.96–4.12; *p* = 0.064) could be obtained in the multivariate analysis for GFS, but lower CD34 count (HR = 3.11, 95% CI: 1.43–6.75; *p* = 0.004), PGF (HR = 11.32, 95% CI: 4.56–28.11; *p* < 0.001), and SGF (HR = 5.45, 95% CI: 2.33–12.79; *p* < 0.001) evidently increased the risk of mortality. The lower TNC count did not influence the RFS (HR = 1.85, 95% CI: 0.90–3.82; *p* = 0.093) in multivariate analysis, but conditioning regimens (HR = 0.44, 95% CI: 0.19–1.00; *p* = 0.05), lower CD34 count (HR = 3.09, 95% CI: 1.41-6.75; *p* = 0.005), primary graft failure (HR = 14.18, 95% CI: 5.67–35.43; *p* < 0.001), and secondary graft failure (HR = 7.97, 95% CI: 3.33-19.05; *p* < 0.001) were independent risk factors of RFS.

The main infectious complications observed after transplant were culture proven bacterial infections in 32.3% (*n* = 42) patients altogether. Fifty-four percent (*n* = 23/42) and 45.2% (*n* = 19/42) patients in the Flu/Cy and ATG/Cy groups acquired bacterial infections. CMV antigenemia occurred in 56.2% (*n* = 73) patients, and it was more frequently observed in the Flu/Cy group, 75.3% (*n* = 55/73), as compared to the ATG/Cy group, 24.7% (*n* = 18/73). BKV reactivation was observed in 2 patients in the Flu/Cy group. The radiological evidence of fungal infection was reported in 3.8% (*n* = 5) patients altogether, in which 60% (*n* = 3/5) patients were in the Flu/Cy group while 40% (*n* = 2/5) patients were in the ATG/Cy group. Hemorrhagic cystitis occurred in 16.2% (*n* = 21). The main cause of death was sepsis in our patients; however, the incidence of infectious complications did not differ between the two conditioning groups.

## 4. Discussion

Graft failure and graft versus host disease remain the main concerns in aplastic anemia, both adversely affecting the outcome [8]. Various attempts have been made to optimize the conditioning regimens to accomplish adequate engraftment with minimal regimen-related toxicity [9]. Antithymocyte globulin and cyclophosphamide combination has been considered the gold standard conditioning in aplastic anemia. The addition of ATG to cyclophosphamide has been shown to promote neutrophil engraftment and resulted in a lower incidence of GvHD and improved overall survival in a nonrandomized study [10]. Another study by Storb et al. reported improved engraftment and prolonged survival in patients receiving the ATG/cyclophosphamide regimen. It is uncertain whether the addition of ATG or advancement in supportive care resulted in the superior outcome [11]. On the contrary, a comparative trial of cyclophosphamide alone conditioning versus ATG/cyclophosphamide by the International Bone Marrow Transplant Registry, involving 134 patients, did not prove any benefit of using ATG, exhibiting similar rates of acute and chronic GvHD and hematopoietic recovery in both the conditioning regimens [12].

Recently, fludarabine-based conditioning regimens have largely demonstrated to augment the sustainability of donor graft without expanding the risk of complications [13]. Fludarabine is combined with low-dose cyclophosphamide to intensify immunosuppression and reduce toxicity [14, 15]. The British Committee for Standards in Haematology also recommend flu-based conditioning regimens in adult aplastic anemia cases [16]. The Japanese aplastic anemia working party reported 83% failure-free survival among children < 16 years of age using Flu/Cy conditioning therapy [17]. A Chinese study group reported the experience of using the PBSC source in 46 adult severe aplastic anemia patients who underwent matched-related and unrelated transplants using fludarabine-based conditioning therapy. Grade II to IV acute GvHD occurred in 4 out of 46 patients (8.7%) in this study [18].

In this study, the Flu/Cy-based conditioning manifested comparable outcomes, conferring to the speculation; the OS, GFS, and RFS were similar and so were the incidences of PGF and SGF. Flu/Cy conditioning was well tolerated by patients in this study with very low regimen-related toxicity, either related to GvHD, infection, or venoocclusive disease, observed. Cyclophosphamide was given at 200 mg/kg to prevent late graft failure reported with low-dose cyclophosphamide in Flu/Cy-based conditioning regimens. The European Group for Blood and Marrow Transplantation (EBMT) initially recommended cyclophosphamide at 40 mg/kg in combination with fludarabine and ATG; however, a high incidence of graft failure was observed in a study, which compelled the investigators to increase the cyclophosphamide dose to 120 mg/kg [19]. Another prospective study compared four different doses of cyclophosphamide used in combination with fludarabine and ATG, and the results showed that the optimal dose of cyclophosphamide was 100 mg/kg at which minimal conditioning-related toxicity was noticed; however, the incidence of late graft failure remained high in this study arm [20]. This study suggests that cyclophosphamide can be safely used at 200 mg/kg in combination with fludarabine for aplastic anemia with minimum regimen-related toxicity.

Majority of patients in both the study arms received peripheral blood as the source of stem cells. The use of PBSC is associated with early engraftment and reduced rate of rejection in patients with aplastic anemia who have been heavily transfused [5, 21]. Strikingly, in this study, the GFS was similar in both the conditioning groups when peripheral blood was compared to bone marrow graft. Even though PBSC was the main source of graft in this study, the incidence of grade III–IV acute GvHD was lower (9.0%); this finding is supported by the data published both from the CIBMTR and from the EBMT [22, 23]. Though 9% of patients developed cGvHD in Flu/Cy group, it was limited in most of the patients. This incidence of cGvHD in this study is better than the data from CIBMTR, which reported rates of 43% with the use of PBSC [22].

The median age of patients in this study was 16 years, and 38.5% of the patients in this study were >18 years of age at the time of transplant. In this study, we did not find any significant impact of age on OS, GFS, or RFS. This again suggests better tolerance of Flu/Cy conditioning in young as well as adult patients. This finding is in contrast to what has been published previously. Age is considered a key predictor of outcome as the risk of morbidity and mortality arising from transplant increases with age [24, 25]. Regarding MSD transplant, the Center for International Blood and Marrow Transplant Research (CIBMTR) study of 1307 patients identified significant impact of age on OS with 82%, 72%, and 53%, OS in <20 years, 20 to 40 years, and >40 years, respectively [26]. Transplant-related deaths were mainly attributable to sepsis secondary to bacterial or fungal infections. Optimization of antimicrobial prophylaxis and reducing the time to transplant from diagnosis has been associated with reduction in early mortality over the last few years.

Our study has some limitations like its retrospective nature, but the data shows that Flu/Cy-based conditioning was well tolerated by the patients with similar OS, GFS, and RFS as observed with ATG/Cy; hence, it should be considered an alternative conditioning regimen for developing countries where aplastic anemia is prevalent, and due to scarce health care resources, transplant cannot be offered to poor patients. This observation needs to be validated on large scale with prospective multicenter trials.

## Figures and Tables

**Table 1:** Baseline characteristics of acquired aplastic anemia patients (*n* = 130).

Median (interquartile range) (range)		
Age (years)		16 (11-20) (3-48)
Time duration from diagnosis till transplant (months)		3 (2-4) (1–8)
Time duration from transplant till follow-up (months)		30 (8-55) (0-98)
		*n* (%)
Age groups (years)	<18 years	77 (59.2)
	≥18 years	53 (40.8)
Gender	Male	89 (68.5)
	Female	41 (31.5)
Conditioning	Flu/Cy	88 (67.7)
	ATG/Cy	42 (32.3)
Cyclosporin	Yes	114 (87.7)
	No	16 (12.3)
Graft source	PB	81 (62.3)
	BM	34 (26.2)
	PB/BM	15 (11.5)

**Table 2:** Distribution of Flu/Cy verses ATG/Cy with transplant characteristics (*n* = 130).

		Flu/cy	ATG/cy	*p* value
		*n* = 88	*n* = 42	
		Median	Median	
		(IQR)	(IQR)	
Age (years)		17 (12-24)	12 (8-18)	0.001^∗^
Time duration from diagnosis till transplant (months)		3 (2-4)	3 (2-4)	0.267
Time duration from transplant till follow-up (months)		30 (8-49)	24 (3-64)	0.813
		*n* (%)	*n* (%)	
Gender mismatch	Female to male	13 (14.8)	6 (14.3)	0.941
	Same	75 (85.2)	36 (85.7)	
Cyclosporine use	Yes	79 (89.8)	35 (85.3)	0.392
	No	9 (10.2)	7 (16.7)	
Graft source	PB	58 (65.9)	23 (54.8)	0.351
	BM	22 (25.0)	12 (28.6)	
	PB/BM	8 (9.1)	7 (16.6)	
Primary graft failure	Yes	5 (5.7)	8 (19.0)	0.027^∗^
	No	83 (94.3)	34 (81.0)	
Secondary graft failure	Yes	9 (10.2)	7 (16.7)	0.392
	No	79 (89.8)	35 (83.3)	
GvHD	Yes	16 (18.2)	2 (4.8)	0.038^∗^
	No	72 (81.8)	40 (95.2)	
Acute GvHD	Yes	7 (8.0)	1 (2.4)	0.436
	No	81 (92.0)	41 (97.6)	
Chronic GvHD	Yes	8 (9.1)	2 (4.8)	0.499
	No	80 (90.9)	40 (95.2)	
Relapse	Yes	8 (9.1)	1 (2.4)	0.270
	No	80 (90.9)	41 (97.6)	

^∗^ *p* value calculated by using Mann–Whitney *U* test, chi-square test, and Fisher's exact test.

**Table 3:** Univariate analysis of influenced factors for overall survival (OS), GvHD-free survival (GFS), and relapse-free survival (RFS).

Variable		OS		GFS		RFS	
		Median (IIQR) *n* = 96	*p* value	Median (IQR) *n* = 82	*p* value	Median (IIQR) *n* = 92	*p* value
Time diagnosis to transplant (months)		3 (2-4)	0.265	3 (2-4)	0.434	3 (2-4)	0.242
		n (%)		n (%)		n (%)	
Age group	<18years	58 (60.4)	0.687	51 (62.2)	0.539	55 (59.8)	0.839
	≥18 years	38 (39.6)		31 (37.8)		37 (40.2)	
Gender	Male	67 (69.8)	0.668	69 (84.1)	0.507	64 (69.6)	0.668
	Female	29 (30.2)		23 (28.0)		28 (30.4)	
Gender mismatch	Female to male	15 (15.6)	0.779	13 (15.9)	0.774	14 (15.2)	0.778
	Same	81 (84.4)		60 (64.5)		78 (84.8)	
Conditioning	Flu/Cy	67 (69.8)	0.401	55 (67.1)	0.579	64 (69.6)	0.521
	ATG/Cy	29 (30.2)		27 (32.9)		28 (30.4)	
Disease category	SAA	88 (91.7)	0.110	74 (90.2)	0.103	85 (92.4)	0.188
	VSAA	8 (8.3)		8 (9.8)		7 (7.6)	
Cyclosporine use	Yes	87 (90.6)	0.126	74 (90.2)	0.134	83 (90.2)	0.133
	No	9 (9.4)		8 (9.8)		9 (9.8)	
CD34	<2.6	11 (11.5)	0.008^∗^	9 (11.0)	0.008^∗^	11 (12.0)	0.010^∗^
	≥2.6	85 (88.5)		73 (89.0)		81 (88.0)	
TNC	<3.3 × 10^8^/kg	17 (17.7)	0.050^∗^	14 (17.1)	0.049^∗^	16 (17.4)	0.001^∗^
	≥3.3 × 10^8^/kg	79 (82.3)		68 (82.9)		76 (82.6)	
Graft source	PB	62 (64.6)	0.427	53 (64.6)	0.498	60 (65.2)	0.443
	BM	25 (26.0)		21 (25.6)		23 (25.0)	
	PB/BM	9 (9.4)		8 (9.8)		9 (9.8)	
Primary graft failure	Yes	3 (3.1)	<0.001^∗^	3 (3.7)	<0.001^∗^	2 (2.2)	<0.001^∗^
	No	93 (96.9)		79 (96.3)		90 (97.8)	
Secondary graft failure	Yes	6 (6.2)	0.001^∗^	6 (7.3)	0.006^∗^	4 (4.3)	<0.001^∗^
	No	90 (93.8)		76 (92.7)		88 (95.7)	

^∗^ *p* value calculated by using chi-square test and Fisher's exact test.

**Table 4:** Multivariate analysis for factors affecting overall survival, GvHD-free survival, and relapse-free survival.

		OS			GFS			RFS		
		HR	95% CI	*p* value	HR	95% CI	*p* value	HR	95% CI	*p* value
Conditioning	Flu/Cy	1			1			1		
	ATG/Cy	0.48	0.22-1.10	0.084	0.47	0.21-1.06	0.072	0.44	0.19-1.00	0.050^∗^
TNC	≥3.3 × 10^8^/kg	1			1			1		
	<3.3 × 10^8^/kg	1.97	0.95-4.07	0.066	1.99	0.96-4.12	0.064	1.85	0.90-3.82	0.093
CD34	≥2.6	1			1			1		
	<2.6	3.18	1.46-6.94	0.003^∗^	3.11	1.43-6.75	0.004^∗^	3.09	1.41-6.75	0.005^∗^
Primary graft failure	No	1			1			1		
	Yes	13.22	5.30-33.02	<0.001^∗^	11.32	4.56-28.11	<0.001^∗^	14.18	5.67-35.43	<0.001^∗^
Secondary graft failure	No	1			1			1		
	Yes	6.45	2.74-15.17	<0.001^∗^	5.45	2.33-12.79	0.001^∗^	7.97	3.33-19.05	<0.001^∗^

^∗^Multivariate Cox regression analyses were applied for hazard ratios. A multiple Cox regression analysis was run to predict OS, GFS, and RFS from conditioning, TNC, CD34, primary graft failure, and secondary graft failure.

## Data Availability

The datasets used and/or analyzed during the current study are available from the corresponding author on reasonable request.
